# How to Choose the Optimal Surgical Strategy to Predict and Prevent LFSS Following Liver Transplantation?

**DOI:** 10.3389/ti.2022.10805

**Published:** 2022-09-05

**Authors:** Diao He, Xingyu Pu, Li Jiang

**Affiliations:** ^1^ Key Laboratory of Transplant Engineering and Immunology, Laboratory of Liver Transplantation, Frontiers Science Center for Disease-Related Molecular Network, West China Hospital, Sichuan University, Chengdu, China; ^2^ Department of Liver Surgery, West China Hospital, Sichuan University, Chengdu, China; ^3^ Department of General Surgery, West China Tianfu Hospital, Sichuan University, Chengdu, China

**Keywords:** LFSS, prediction, prevention, adult, reduced-size liver transplantation

We appreciate the positive feedback that Zhou GP and his colleagues provided on our article, “A Novel Strategy for Preventing Posttransplant Large-For-Size Syndrome in Adult Liver Transplant Recipients: A Pilot Study” (1). Their article raised several concerns on our published article. We are grateful to the Editor for allowing us to respond to these comments.

It is crucial to match donor and recipient sizes appropriately to prevent Large-for-Size Syndrome (LFSS). A valuable idea presented by Zhou et al. is the incorporation of graft morphological parameters, particularly the anteroposterior (RAP) vertical distance and the longest horizontal distance, into the LFSS indicator (2). By combining the morphological parameter of graft, graft-recipient weight ratio (GRWR) and graft weight (GW)/RAP, it is possible to more accurately indicate the need for reduction of the right graft (3).

The point is how to measure the morphological parameter of graft using an appropriate method. As of today, computed tomography (CT) scan is the most accurate method to measure the right RAP vertical distance and the largest horizontal distance of grafts in living donor liver transplantation (LDLT) (4). However, Donation after Citizens Death (DCD) donors need to receive treatment in the intensive care unit and should not be moved, which limits the use of CT scans in for measuring graft parameters in deceased donor liver transplantation (DDLT). Doppler ultrasonography can be performed at the bedside, but DCD donors may experience edema in their gastrointestinal tracts during maintenance periods, affecting the accuracy of the measurement results. Alternatively, measurements can be taken during graft procurement period, which has the advantage of being done under naked eye conditions. In view of the fact that the graft does not have blood filling *in vitro*, the *ex vivo* measurement value is smaller than the actual one *in vivo*. For a closer match between *in vitro* and *in vivo* measurement values, we propose to combine several transplant centers and develop a new calculation formula with a large sample size.

Paterno et al. recently proposed a new solution, “bilateral marginal costotomy,” for rescuing a liver transplant recipient from severe graft compression caused by bilateral narrow rib cages after temporary abdominal closure failed (5). Yet, this method is more likely to be a salvage measure for donor-recipient matching fails than a conventional treatment since the thoracic cavity needs to be changed, increasing the risk of postoperative complications. In contrast, according to our observations using the HuaXi-eRPS technique, all recipients had intact hepatic arteries, hepatic veins, and biliary tracts as well as good blood supply without any biliary complications. Thus, HuaXi-eRPS under the existing conditions should be considered a safe and effective procedure for the prevention of posttransplant LFSS. With the advancement of technology, we will also try new detection methods and incorporate new predictive indicators in order to make more effective control strategies for posttransplant LFSS.

## Data Availability

The original contributions presented in the study are included in the article/supplementary material, further inquiries can be directed to the corresponding author.
